# Reduced Neonatal Mortality in Meishan Piglets: A Role for Hepatic Fatty Acids?

**DOI:** 10.1371/journal.pone.0049101

**Published:** 2012-11-14

**Authors:** Hernan P. Fainberg, Katherine Bodley, Jaume Bacardit, Dongfang Li, Frank Wessely, Nigel P. Mongan, Michael E. Symonds, Lynne Clarke, Alison Mostyn

**Affiliations:** 1 School of Veterinary Medicine and Science, University of Nottingham, Leicestershire, United Kingdom; 2 School of Computer Science, University of Nottingham, Nottingham, United Kingdom; 3 School of Biosciences, University of Nottingham, Leicestershire, United Kingdom; 4 Early Life Nutrition Research Unit, University of Nottingham, Nottingham, United Kingdom; 5 Department of Agricultural Sciences, Imperial College Wye, Wye, Kent, United Kingdom; University of South Alabama, United States of America

## Abstract

The Meishan pig breed exhibits increased prolificacy and reduced neonatal mortality compared to commercial breeds, such as the Large White, prompting breeders to introduce the Meishan genotype into commercial herds. Commercial piglets are highly susceptible to hypoglycemia, hypothermia, and death, potentially due to limited lipid stores and/or delayed hepatic metabolic ability. We therefore hypothesized that variation in hepatic development and lipid metabolism could contribute to the differences in neonatal mortality between breeds. Liver samples were obtained from piglets of each breed on days 0, 7, and 21 of postnatal age and subjected to molecular and biochemical analysis. At birth, both breeds exhibited similar hepatic glycogen contents, despite Meishan piglets having significantly lower body weight. The livers from newborn Meishan piglets exhibited increased C18∶1n9C and C20∶1n9 but lower C18∶0, C20∶4n6, and C22∶6n3 fatty acid content. Furthermore, by using an unsupervised machine learning approach, we detected an interaction between C18∶1n9C and glycogen content in newborn Meishan piglets. Bioinformatic analysis could identify unique age-based clusters from the lipid profiles in Meishan piglets that were not apparent in the commercial offspring. Examination of the fatty acid signature during the neonatal period provides novel insights into the body composition of Meishan piglets that may facilitate liver responses that prevent hypoglycaemia and reduce offspring mortality.

## Introduction

Although genetic similarities exist between oriental Meishan and European commercial pigs such as the Large White, there are significant morphological and physiological differences which are the consequence of intense phenotypic selection [1]. Meishan offspring are typically from larger litters, lighter at birth, slower growing and have increased intramuscular fat content in comparison to domestic European pigs [2]. However, Meishans exhibit very low rates of neonatal mortality in comparison to their European commercial counterparts [2], [3], [4]. Previous studies have described important differences in endocrine and physiological factors during gestation which regulate fetal growth and thereby improve neonatal survival [5], [6]. For instance, by late gestation, the Meishan placenta is more vascular and therefore adapted to meet the slower rate of fetal growth, in a larger number of fetuses, in comparison to European breeds [3], [5]. Furthermore, Meishan offspring typically exhibit little variation in birth weight which may limit competition between littermates, and potentially be a major survival advantage [3]. In contrast, the offspring of commercial large white pigs, show a much greater difference in birth weight and often produce one or more low birth weight piglets, indicative of growth restriction during gestation [3]. These piglets (typically described as “runts”) may then be unable to feed as regularly at birth, due to increased competition from their normal sized litter-mates and are therefore more susceptible to hypoglycemia, hypothermia and death [7]. One important factor contributing to neonatal hypoglycemia in Large White offspring is limited reserves of hepatic fat and glycogen [8].

After birth, important physiological processes such as thermoregulation and growth require a rapid metabolic adaptation to increase the rate of fatty acid and lactate oxidation, a function that occurs primarily in the liver [9], [10]. This produces a high requirement for fatty acids that is provided by maternal colostrum [11], [12]. However, the newborn pig has a limited capacity for lipid oxidation and formation of ketone body production, making them highly dependent on glycolysis for meeting energy demands [13], [14], [15]. Despite these difficulties, there are specific fatty acids with a greater oxidative capacity, including oleic acid (C18∶1n9C) and shorter fatty acid chains (C <14) [13]. These are oxidized more rapidly than saturated fatty acids, such as palmitic (C16∶0) and stearic acid (C18∶0), particularly in the presence of glycogen [13]. In addition, other families of lipids, known as n-3 and n-6, exceed the typical role of energy storage molecules and participate in a variety of physiological processes, such as inflammation, and skeletal as well as cardiac muscular growth. In particular, arachidonic acid (C20∶4n4) and docosahexaenoic acid (C22∶6n3) are known to have significant anabolic effects during gestation and in the newborn [16], [17], [18]. Cellular uptake of fatty acid induces both oxidative and catalytic mechanisms that are regulated by enzymes, including acetyl-coenzyme A (*CoA*) carboxylase, fatty acid synthase (*FAS*), stearoyl-CoA desaturase (*SCD*)-1 and the elongation of very long chain fatty acids (*ELOVL*)-6, which are all active in the newborn liver but the effect of breed on the lipid profile and handling is not known [19].

The liver has a major role in regulating glucose production, glycogen synthesis and lipid handling and is likely to be a primary determinant in the increased survival of Meishan compared with commercial piglets especially as hypoglycaemia is a significant contributor to mortality [7]. We therefore utilized a lipidomic approach in order to compare differences in hepatic lipid pathways over the first three weeks of newborn life between these breeds. This techniques has provided new insights into our understanding of complex lipid metabolic pathways in humans [20] and animals [21]. We now report the first comparative lipidomic analysis of the liver between two porcine breeds on samples taken during the first three weeks of postnatal life.

## Materials and Methods

### Ethics Statement

All operative procedures and experimental protocols had the required Home Office approval as designated by the Animals (Scientific Procedures) Act (1986) and had local ethical approval (Imperial College Wye).

### Animals

Thirteen Meishan sows and seventeen commercial sows (Large White cross) of known mating date and parity were used in this study. All the animals were kept on the same commercial unit. All piglets were weighed approximately 4 hours after birth and the closest to the median weight were randomly assigned to be tissue sampled on day 0 (Meishan n = 5; commercial n = 5), 7 (Meishan n = 6; commercial n = 6) and 21 (Meishan n = 5; commercial n = 5) of postnatal age. The ratio of males and females within each group for the 0 and 21 day old groups was 2∶1 and 1∶1 for the 7 days group of each breed. On each assigned day, the offspring were humanely euthanized with an overdose of barbiturate anesthetic (200 mg/Kg: Euthatal: RMB Animal Health, Stoke, UK). Liver weight was noted immediately and samples were frozen in liquid nitrogen and subsequently stored at −80°C until analysis. All the homogenizations were performed using a gentleMACS™ closed homogenizer (Milteny Biotec Ltd., Surrey, UK).

### Biochemical Analysis

#### Determination of liver glycogen content

Glycogen content was determined using the method developed by Dalrymple and Hamm [22]. The concentration of glucose released from this reaction was measured by a colorimetric commercial assay (Randox Ltd., Country Antrim, UK) and the results were expressed as a concentration of milligrams of glycogen per gram of tissue dissected.

#### Determination of liver triglyceride content

Hepatic lipid content was assessed using the Folch method; briefly, 0.5 g hepatic tissue was homogenized in a total volume of 10 ml cold chloroform/methanol (2∶1) and agitated for 20 minutes at room temp followed by phase separation through centrifugation at 2000 rpm for 10 minutes [23].The concentration of triglycerides obtained from this reaction was determined by colorimetric assay (Randox Ltd., Country Antrim, UK) and the results were expressed as a concentration of milligrams of triglycerides per gram of tissue dissected.

#### Determination of liver phospholipid composition

Gas-chromatography (GC) was used to determine the fatty acid composition in phospholipids. The chloroform phase was evaporated by applying a nitrogen stream and then 2 ml of hexane was used to re-dissolve each sample. Samples were transesterified by the method of Christie [24] and modified by Chouinard *et al*. [25]. Briefly, 40 µl of methyl acetate was added to the phospholipid sample followed by vortexing. Then 40 µl of methylation reagent (0.9 ml of 30% sodium methoxide in 4.1 ml methanol; Fisher Scientific Ltd. Loughborough, UK) was added to each reaction. The mixture was vortexed and allowed to react for 10 minutes at room temperature and then 60 µl of termination reagent (0.2 g oxalic acid in 6 ml diethyl ether) was added followed by vortexing. 200 mg of calcium was added and allowed to stand for 1 hour to absorb the moisture. The samples were centrifuged for 5 minutes at 3400 x g and the supernatant transferred to a gas chromatography vial and used directly for gas chromatography. The fatty acid methyl esters were then injected (split ratio 50∶1) into gas chromatograph (GC 6890; Agilent technologies Ltd, Stockport, UK). Separation of fatty acid methyl esters was performed with a Varian CP-Sil 88 (Crawford Scientific™ Ltd., Strathaven, UK) capillary column with hydrogen as carrier gas. Oven temperature was programmed from 59°C to 100°C at 8°C per min, then to 170°C at 6°C per minute and held for 10 minutes, and then to 240°C at 3°C per min and held for 10 min. The temperature of the injector and detector were set at 255°C and 250°C. The fatty acid methyl esters were identified by comparing the retention times with a fatty acid methyl esters standard mixture (Sigma-Aldrich Co LLC, Gillingham, UK) and the area percentage in moles were used for the statistical analysis.

A total of 37 fatty acids were analyzed in this study and included: saturated fatty acids: C4∶0, C6∶0, C8∶0, C10∶0, C11∶0, C12∶0, C14∶0, C15∶0, C16∶0, C17∶0, C18∶0, C20∶0, C21∶0, C22∶0, C23∶0, C24∶0. Monousaturated fatty acids: C14∶1, C15∶1, C16∶1, C17∶1, C20∶1, C24∶1. Polysaturated fatty acid- Omega (n)-3: C18∶3n3, C20∶3n3, C20∶5n3, C22∶6n3. Omega (n)-6: C18∶2n6t, C18∶2n6, C20∶3n6, C20∶4n6. Omega (n)-9: C18∶1n9t, C18∶1n9C, C20∶1n9.

### Gene Expression Analysis

Total RNA was extracted from 40 mg liver using a commercially available kit (Qiagen Ltd., Crawley, UK), which included a DNA purification step. The amount and purity of the extracted RNA was determined using a Nanodrop (Thermo Fisher Scientific Ltd. Leicester, UK). Total RNA (1 µg) was reverse transcribed using a thermocycler (Thermo Fisher Scientific Ltd. Leicester, UK). Quantitative PCR (qPCR) was performed using a Roche Lightcycler 480 and SYBR technology (Roche, Burges Hill, UK). For quantification of the gene expression we used 18s RNA abundance as a housekeeping gene for the normalization of the mRNA expression. Data were analyzed using the ΔΔ^CT^ method [26].

The qPCR primers were designed based on known porcine sequences published on Genbank using online software (Primer3), on intra-exonic boundaries where possible. A standard curve was included and the samples were run in duplicate as well as having the appropriate positive and negative controls. The primers used were purchased from Eurofins MWG Operon GmbH (Ebersberg, Germany) and validated as described in previous publications [27]. The following porcine specific oligonucleotide forward (F) and reverse (R) primers used were as follows:

*FAS* F: 5′-CGGCTCACACACCTTCGT-3′ and R: 5′-TGCTCCATGTCGGTGAACT-3′

*CoA* F: 5′-ACCAGGCAACTGAGGAACAG-3′ and R: 5′-TCCAAGCCTCGAAGATGAGT-3′

*SCD-1* F: 5′-CTGGTTTCATTGGGAGCTGT-3′ and R: 5′-CGCTGGCAGAATAGTCATAGG-3

*ELVOL-6* F:5′-CCAATGGATGCAGGAAAAACT-3′ and R: 5′-GCCAAAGATAAAGGCAGCAT-3′.

The primers for 18s have previously been published [27].

### Data Analysis

#### Statistical analysis

Data obtained from the liver phospholipid composition was filtered of poorly measured fatty acids (defined as fatty acids with percentiles below 0.01%). All data were evaluated using SPSS 16.0 (SSPS® Inc. Chicago, USA) for windows applying General Linear Model analysis and adjusted by Bonferroni correction. Only differences between postnatal age and breed, including a correction for sex, with p<0.05 were considered as significant. In all the cases, the results are given as mean ± SEM.

#### Unsupervised analysis

The R-package gplots was used for hierarchical clustering analysis of two lipid profiles to identify groups within each breed independently. Clustering results are presented as dendograms and heatmaps. We used the class discovery module of the public online software ArrayMining.net [28] (http://arraymining.net/), originally developed for microarray data to assess the clustering results obtained from various clustering algorithms. Additionally, Matlab (MathWorks® Inc. Natick, USA) was used for the principal component analysis (PCA).

#### Machine learning analysis

We have also processed the lipid profile data using machine learning (ML) techniques to measure the predictability of these samples and identify key variables involved in these predictions; these techniques have been applied to a diverse range of data sets by the authors [29], [30]. To this aim we have used an analysis procedure that was recently applied to identify novel regulators of seed germination from transcriptomics data [31]. The core of this analysis procedure is a rule-based machine learning system called BioHEL [32] (http://icos.cs.nott.ac.uk/software/biohel.html). BioHEL automatically generates, from a set of samples, a series of production rules (an example of a high-quality rule set is available in the results section) that contain human-readable explanations of why a given sample belongs to a class (e.g. commercial vs. Meishan pigs, or 0-day samples vs. 7/21 day samples). To evaluate the prediction capacity of BioHEL we have employed the leave-one-out procedure, where we generate a rule set using all samples except one and validate the prediction capacity of the generate rule set on the remaining sample. This procedure is repeated as many times as samples, using each time a different one as test. We have evaluated the prediction capacity of several scenarios: Meishan vs. Commercial (tested separately for each of the three age groups), 0 days vs. 7 days vs. 21 days (separately for Meishan and Commercial pigs) and two variants of the latter: 0 days vs. 7+ days and 7- days vs. 21 days. In total we are evaluating 9 scenarios.

## Results

### Body Growth and Liver Characteristics

**Figure 1:**
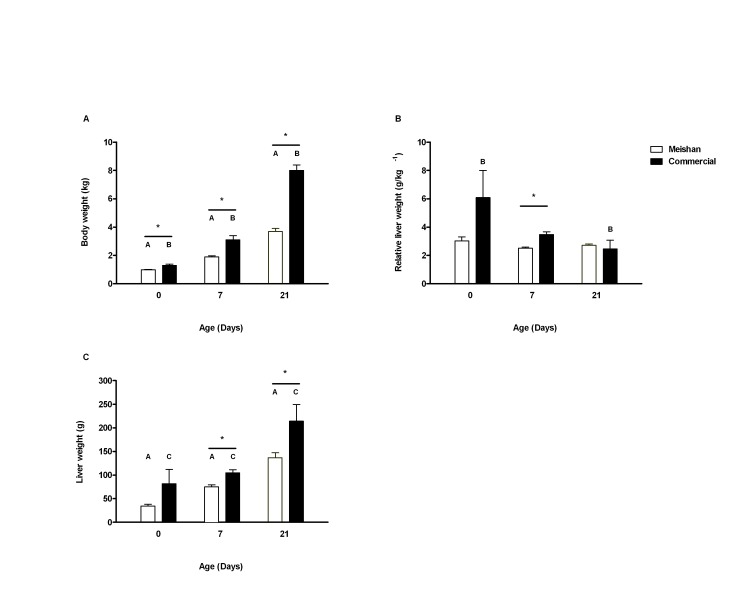
Body and liver weight with age and breed. Influence of breed on early growth in Meishan (open bars) and commercial (closed bars) piglets during the first 21 days postnatal life. (A) Changes in body weight, (B) Changes in liver weight and (C) Relative liver weight. A; p<0.05: statistical difference between the Meishan breed age groups, B; p<0.05: Statistical difference between the commercial age groups, C; p<0.05: Statistical difference between the 0 and 7 day old in comparison to 21 days old commercial breed group and *; p<0.05: Statistical difference between the same postnatal age groups. Bar graphs illustrate means ± SEM.

As previously observed by Mostyn *et al*., [33] commercial piglets were significantly heavier than the Meishan group throughout the study (Figure 1A). In both breeds, liver weight increased with age, however, this was only significantly different between the Meishan and commercial groups after one week of postnatal age (Figure 1B). Interestingly, despite these increases in liver weight, there is a clear disparity in the rate of liver growth in commercial piglets during the postnatal period in relation to the increase in global body weight and in relation to the Meishan group. Thus, at 7 days of age the commercial offspring had a lighter liver relative to their body weight compared to the Meishan group, this reduction in liver growth persisted until 21 days of age when they had a relatively smaller liver compared to the measurements observed at birth (Figure 1C).

**Figure 2:**
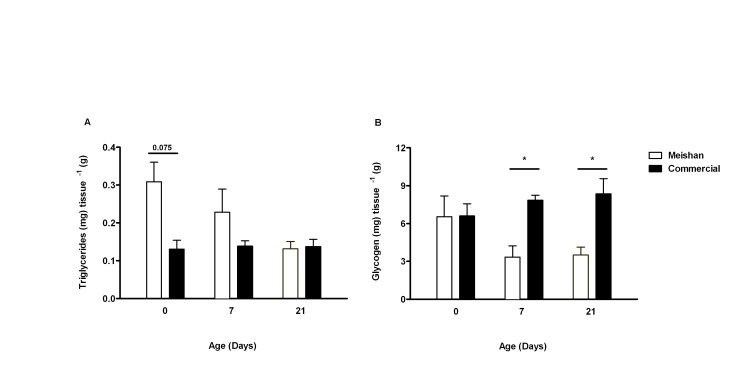
Liver composition between age and breed. The effect of breed and postnatal age in liver composition between Meishan (open bars) and commercial (closed bars) piglets: (A) Triglyceride content and (B) glycogen content. *; p<0.05: statistical difference between the same postnatal age groups. Bar graphs illustrate means ± SEM.

At birth, the Meishan group had a trend for higher hepatic triglyceride content, which subsequently declined with age (Figure 2A). A significant reduction in hepatic glycogen was observed up to day 21 in the Meishan piglets (Figure 2B).

### Effects of Breed and Age on Hepatic Fatty Acid Composition

**Figure 3:**
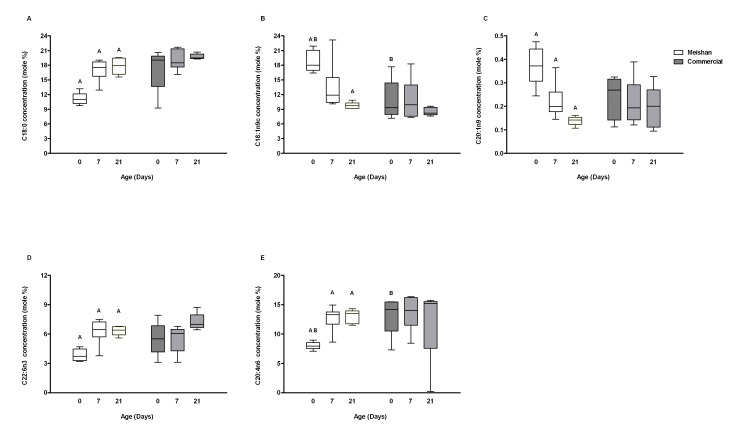
Fatty acid profiles. Differences of the major fatty acids in the livers of Meishan (open bars) and commercial (grey bars) piglets over the first 21 days of postnatal life. (A) stearic acid, (B) heptadecanoic acid, (C) oleic acid, (D) eicosenoic acid (E) arachidonic acid, (F) docosahexaenoic acid. A; p<0.05: statistical difference between the 0 and the other age groups within Meishan breed group. B; p<0.05: Statistical difference between the same postnatal age groups. Bar graphs illustrate means ± SEM.

Only five fatty acids analysed during this study showed significant differences between breeds, with the most variable lipid profile observed in the Meishan newborn. Among the two breeds, the proportions of most saturated fatty acids were similar, however, a reduction in the proportion of stearic acid (C18∶0; Figure 3A) was observed on day 0 in the Meishan piglets. Compared with commercial piglets, day 0 Meishans had a significant increase in oleic acid (C18∶1n9C) (Figure 3B). Eicosenoic acid (C21∶0n9) was also increased in day 0 Meishan livers and, as observed with oleic acid, it declined with age (Figure 3C). Among n-6 fatty acids, docosahexaenoic acid (C22∶6n6) was lowest at birth in Meishan pigs, increasing with age and showing similar values at 7 and 21 days of age (Figure 3D), a similar pattern was observed for of the n-3 fatty acid n-3 arachidonic acid (C20∶4n3) (Figure 3E).

### Lipidomic Analyses

**Figure 4:**
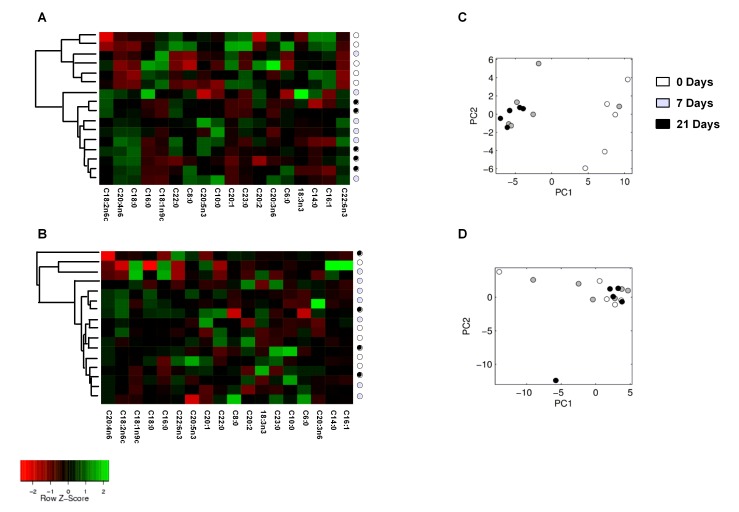
Heat maps and principal component analysis of fatty acid profiles. Heat maps and dendrograms visualizing the hierarchical clustering results (average linkage, Euclidean distance metric) of Meishan (A) and commercial piglets (B). Rows correspond to animals and the corresponding age group is indicated by coloured circles. The fatty acid mole percentiles were transformed to a z-score and indicated by a colour code, with red representing low percentiles and green high percentiles. Principal component analysis (PCA) plots of all Meishan (C) and all commercial piglets (D).

To explore whether the changes in lipid pathways in the liver were unique to the Meishan breed at birth, we performed lipidomic analysis of the most common 18 fatty acid species. To capture all the alterations in lipid profiles, we first performed unsupervised hierarchal cluster analysis for each breed, focusing on lipid species independent of any significant differences. Each column (Figure 4a and 4B) represents a single lipid profile across the sample data set and each row corresponds to a single animal sample. The percentile value of each fatty acid was transformed by centring and scaling values in a column direction. Transformed values are represented by a colour code, with green and red representing a score above, or below the mean percentile, respectively. The intensity of the colour corresponds to the magnitude of the deviation from the mean. Through this analysis, the lipid samples extracted from Meishan piglets were segregated into two independent clusters: one single cluster included all the newborn Meishans in addition to a single 7-day-old offspring and the rest of the age groups were incorporated into a second cluster (Figure 4A). All available clustering methods of the class discovery module of the Arraymining.net tool [28] generated these two clusters as their best scoring clustering result for the Meishan piglets.

Interestingly, analysis of the commercial piglets regardless of age was unable to identify any substantial age-based cluster (Figure 4B). This is consistent with the results from the class discovery module: all the methods identified as their best scoring clustering resulted in a large cluster with 13 piglets from all ages, with the remaining three piglets in two small clusters of size composed by different age piglets. Classical standardization of samples gave the same clustering results for both breeds.

**Figure 5:**
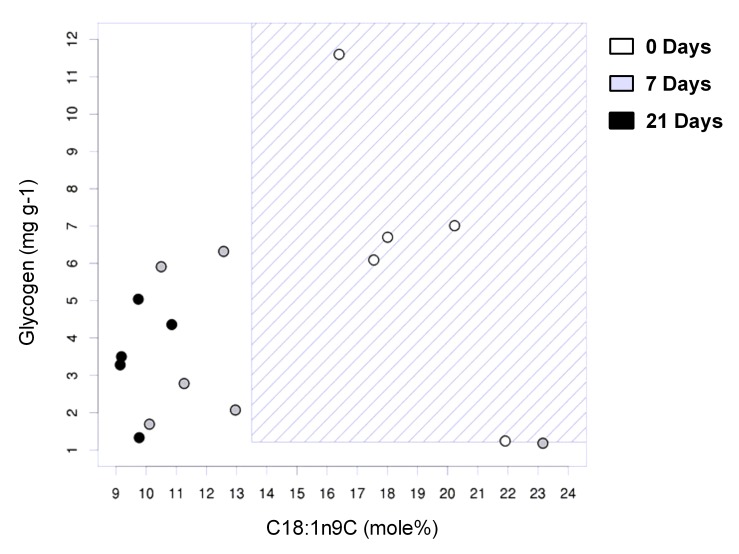
Graphical representation of the interaction between glycogen and oleic acid in Meishan piglets. Graphical representation of the rule set generated by BioHEL to separate between newborn and 7+ day samples in Meishan pigs based on the measurements for C18∶1n9c and glycogen. Each Meishan offspring is represented by a circle and is colour coded as indicated. The marked area corresponds to the parameters that are included in this rule.

Similar results for the unsupervised analysis of the two breeds were obtained by principal component analysis (PCA). Plotting the first two principal components shows a clear separation of the newborn Meishan piglets (Figure 4C). For the commercial group, however, the PCA plot does not show a separation of any group (Figure 4D), suggesting that the changes in lipid metabolism at birth are highly heterogeneous in this breed. The first two components explain 90.9% and 87.3% of the total variance within the two lipid datasets of the Meishan and commercial piglets, respectively. Next, we calculated the association between the different fatty acids and the ability of the liver to produce glycogen by employing the BioHEL machine learning system, which was able to predict developmental outcome and differentiate the newborn from the 7 and 21 day old samples in Meishans with an accuracy of 81.25%. For this scenario BioHEL was able to generate a very simple rule set (Figure 5) with just two rules and using only glycogen and C18∶1n9c. This can be summarised as follows:

**If** glycogen >1.21(mg/ml) **and** C18∶1n9C >13.5 mole% **predict** age 0.

**Everything else** → **predict** age 7+.

Similar to the results obtained from clustering and PCA data, BioHEL was not able to generate accurate rules to identify the newborn from the Large White breed age groups.

### Expression of Genes Associated with Fatty Acid Uptake, Elongation and Mitochondrial Biogenesis

**Figure 6:**
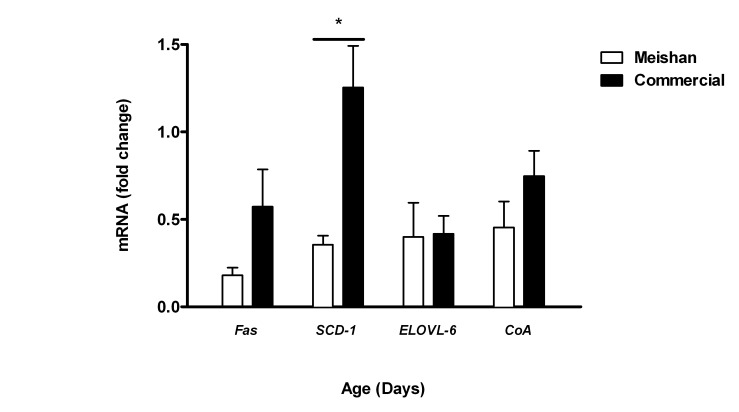
mRNA expression of hepatic metabolic genes between breed and age. mRNA abundance of *FAS*, *SCD-*1, *ELOVL*-6 and *CoA* in the liver of newborn Meishan (open bars) or commercial (closed bars) piglets as determined by real time PCR. Bar graph illustrates means ± SEM with statistically significant differences between groups represented by *p<0.05.

To further assess the changes in liver metabolism at birth among the two breeds, we performed real-time quantitative PCR of the genes involved in several aspects of lipid uptake, transport and metabolism. Hepatic expression of *FAS*, *CoA* and *ELOVL*-6 were similar between breeds. We detected a significant increase of *SCD*-1 n-9 desaturase expression in the liver of new born commercial piglets, compared to Meishan piglets (Figure 6). No differences were observed between breeds at 7 or 21 days (data not shown).

## Discussion

We have demonstrated distinct differences in hepatic lipid composition and substrate regulation between Meishan and Commercial piglets at birth that are likely to reflect divergent developmental profiles between breeds *in utero*. These are likely to contribute to the greater ability of Meishan piglets to survive after birth.

### Meishan Livers Possess a Lipid Profile that could Enable them to be Better Adapted to Withstand Food Shortages after Birth

Despite porcine colostrum being rich in fat [12], newborn piglets are highly susceptible to hypoglycaemia [34], especially those with faster growth rates, such as commercial breeds [4], [10], [35] who maintain hepatic glycogen production (Figure 2B). Maintenance and or production of hepatic glycogen, although necessary to meet sudden nutritional shortages and sustain metabolism, occurs at the expense of plasma glucose and may be a cause of hypoglycaemia in this breed [7]. A major challenge to the newborn pig is to adapt to the substantial changes in dietary composition at birth; switching to a diet low in carbohydrate but high in fat that is the opposite of the *in utero* nutritional environment [36]. During the first weeks of postnatal life there is inefficient oxidation of long-chain fatty acids in neonatal pigs for liver gluconeogenesis, due to an immature capacity of mitochondrial transport [37] and a reduction in pancreatic lipase activity [10]. The lipid profile of the Meishan piglets at birth may offer important insights as to how they evolved to meet this challenge. Although stearic acid (C18∶0) is one of the most abundant dietary fatty acids and can also be obtained through *de novo synthesis* we observed a substantial reduction in the hepatic content for both in newborn Meishan [38]. In contrast, oleic (C18∶1n9C) and eicosenoic acid (C20∶1n9) which are both derived from stearic acid (C18∶0) increased, whereas palmitic acid (C16∶0) (a potential substrate for C18∶0) was unchanged [39]. We propose that the source of both omega-9 fatty acids was from the conversion of stearic acid to n-9 fatty acids during gestation. This may be advantageous for the newborn Meishan as oleic acid can be oxidised or incorporated in triglycerides by the liver more rapidly than stearic acid [13], [38]. In addition, we found a reduction in hepatic gene expression of *SCD-1* in newborn Meishan piglets. This is a key enzyme involved in the desaturation of stearic acid to oleic acid [40] and is suppressed by a diet rich in oleic acid, leading to increased lipid oxidation and glycogen depletion [38]. The developmental “rules” we generated using supervised learning algorithms (BioHEL) were able to recognize the interaction between oleic acid and glycogen as a unique feature for the Meishan breed at birth. In contrast, due to the heterogeneity in the lipid profiles in the commercial group at birth, BioHEL was unable to find such a “rule” for this breed. These observations suggest that the Meishan liver passes through a series of prenatal adaptations that enable the newborn to better adapt to the postnatal nutritional environment, thus increasing their chances of survival.

### Differences in Hepatic Lipid Profile at Birth and the Potential Impact of the Ability of the Meishan Placenta to Select and Limit the Maternal-fetal Exchange of Nutrients

We have identified differences between hepatic composition and lipid profile between Meishan and commercial piglets that may determine neonatal survival [3], [4], [41]. It is known that Meishan sows possess an endocrine network capable of discriminating nutrients that affect placental development and anatomy, reducing fetal growth without compromising neonatal viability [3], [42], [43]. This is in contrast to European breeds, particularly during the last weeks of gestation, whose offspring appear to rely on placental growth to increase nutrient uptake [3], [5]. Therefore, in commercial piglets, this lack of regulation in placental expansion can compromise offspring viability and may be one factor contributing to large variations in size between littermates mediated by competition for nutrients and uterine space [3], [5].

The majority of the fatty acids accumulated in the fetus during gestation are derived via placental transfer [16] for which only trace amounts cross the swine placenta [44]. It is known in humans that specific metabolic pathways exist that facilitate the transfer of C20∶4n6 and C22∶6n3 [16] and both are considered to be essential because of the absence of the enzymes needed for their synthesis. Both n-3 and n-6 fatty acids are necessary for the activation of prostaglandins that induce growth in several tissues, including skeletal muscle and brain [18], [45]. The limited accretion of these fatty acids in the livers of Meishan fetuses may limit fetal growth [18], [45]. In response to starvation, the most common cause of piglet death, liver glycogen stores are rapidly depleted [46] as other energy body reserves, such as fat, represent only between 1 to 2% of the total body weight in European commercial breeds [47]. A greater abundance of triglycerides in the livers of newborn Meishan piglets may provide an initial alternative energy source that is depleted by one week of age. As the liver to body weight ratio in the Meshian piglets remains constant during the first 21 days of life, it is possible to conclude that this organ may play a more important role their survival compared with commercial piglets.

### Changes in Meishan Lipid Profile are the Result of Adaptation to the Postnatal Nutritional Environment

One surprising observation from our study was the substantial differences in the lipid profile between the newborn and older Meishan piglets. These changes in hepatic fatty acid abundance suggest an enhanced ability to respond to the changes in nutrition and are in accordance with previous studies demonstrating enhanced lipid accretion that is indicative of greater metabolic maturity [10].

The rapid changes in hepatic fatty acid content with age could be a response to the comparatively low metabolic need for muscular development and thermogenesis by Meishan piglets during this period [33], [48]. Interestingly, one seven day old Meshian piglet was clustered together with the newborn group; upon further examination this piglet was characterised as failing to grow thereby offering an explanation for its immature lipid profile.

In hepatic tissue, a reduction in mitochondrial number has been observed in newborn commercial pigs, suggesting limited fatty acid oxidation [8], possibly explaining why the lipid composition of adipose tissue resembles the fatty acid profile of colostrum [34], [49]. After the first day of postnatal life carnitine-stimulated oxidation of palmitate increases in the liver and a range of other tissues suggesting the ability of piglets to utilize fat improves with age [50]. The lack of any change in liver lipid profile and triglyceride content with age in the commercial breed suggests mobilisation of lipids from alternative depots [51]. This limitation of liver oxidation appears also to be present in newborn Meishan piglets as the gene expression of key lipogenic enzymes is similar, again highlighting the importance of colostrum in early neonatal survival. Variations in milk lipid and hormonal composition are observed between porcine breeds with Meishan sows producing milk containing more fat and triiodothyronine and less leptin [12], [34], [52].

### Conclusion

This is the first lipidomic study in the neonate that has shown distinct differences in hepatic lipid prolife between two porcine breeds. We detected a difference in the lipid profile ontogeny in the newborn Meishan and were able to identify five different fatty acids that may play important roles in postnatal growth and metabolism as well as potentially revealing important interactions between fetus and placental nutrient exchange during gestation. Further investigations are now needed to evaluate whether the differences in hepatic lipid profiles interact with other tissues during this period of accelerated growth, particularly in adipose tissue and skeletal muscle.
